# Antibacterial and hemocompatibility potentials of nano-gold-cored alginate preparation against anaerobic bacteria from acne vulgaris

**DOI:** 10.1038/s41598-024-57643-5

**Published:** 2024-03-24

**Authors:** Hanan A. Abbas, Ali A. Taha, Ghassan M. Sulaiman, Amer Al Ali, Humood Al Shmrany, Haralambos Stamatis, Hamdoon A. Mohammed, Riaz A. Khan

**Affiliations:** 1 Division of Biotechnology, Department of Applied Sciences, University of Technology, Baghdad, Iraq; 2 Department of Medical Laboratory Sciences, College of Applied Medical Sciences, University of Bisha, 255, 67714 Bisha, Saudi Arabia; 3 Department of Medical Laboratory Sciences, College of Applied Medical Sciences, Prince Sattam Bin Abdulaziz University, 11942 Alkharj, Saudi Arabia; 4 Department of Biological Applications and Technology, University of Ioannina, Ioannina, Greece; 5 Department of Medicinal Chemistry and Pharmacognosy, College of Pharmacy, Qassim University, 51452 Qassim, Saudi Arabia; 6 Department of Pharmacognosy and Medicinal Plants, Faculty of Pharmacy, Al-Azhar University, Cairo, 11884 Egypt

**Keywords:** Gold/alginate nanopreparation, Nanoparticles, Acne, Anaerobic bacteria, *Clostridium innocuum*, *Lactobacillus plantarum*, *Anaerococcus prevotii*, *Peptoniphilus asaccharolyticus*, Hemocompatibility, Biocompatibility, Biotechnology, Nanobiotechnology, Bacterial infection

## Abstract

Acne is a prevalent dermatological disease, with high global incidence, and is a health menace. The current study aimed to isolate and characterize the anaerobic bacteria responsible for the condition. Causes of a total of 70 acne-based bacterium isolates obtained from patients of mild, moderate, and severe acne, 24 were *Clostridium innocuum*, 21 were *Lactobacillus plantarum*, 13 were *Anaerococcus prevotii*, and 12 were *Peptoniphilus asaccharolyticus*. Nearly 69% of males were suffering, while the rest were females at 31%. The 15–30 years old age group was the most affected. The gold/alginate nanoparticles’ nanopreparation (GANPs) produced from chloroauric acid and sodium alginate was an effective treatment against the acne conditions under the experimental conditions. The nanopreparation exhibited significant inhibitory activity against anaerobic bacterial isolates, with a minimum inhibitory concentration of 200 µg/ml for *A. prevotii* and *P. asaccharolyticus,* and 400 µg/ml for *C. innocuum* and *L. plantarum*. The in vitro efficacy of the GANPs on human blood parameters was also assessed. The concurrent results suggested potential antibacterial activity and hemocompatibility of the product, which has promise to be used as a successful antibacterial agent for acne.

## Introduction

Acne, a multifactorial disease^1^, is caused by sebaceous glands malfunctioning leading to sebum production, and follicular keratinization of the pilosebaceous ducts. The ensuing inflammation promotes and increases the growth of inhibiting bacteria that are crucial in the progression of acne. Regulated by several virulence factors and antibiotic resistance, the changes in the sebum quality and quantity during adolescence produce significant effects on the interactions of interspecies microbiome compositions and the host. The actinobacteria, proteobacteria, firmicutes, and bacteroidetes, the four major and common bacterial phyla on the skin^2,3^ engage in the infestation. In this scenario, the topical and systemic antibiotics, which are used to induce cutaneous dysbacteriosis, also respond with side-effects, such as skin irritation, teratogenicity, and resistance to antibiotics. The situation has demanded a potent and secure alternative therapy to manage the acne conditions of individuals of all ages^4^. Nanomedical biotechnology has taken precedence in a number of physiological conditions owing to its outreach, dose control, specificity, and enhanced bioaction. The choice for nanoscale therapeutic agents have gained ground^5^. The nano-scale material’s size-dependent properties make the exhibit unique and favorable for biochemical and biophysical properties, together with physico-chemical properties that are deemed fit for biological and pharmacological activities^6^. The metal nanoparticles (NPs) sourced from gold, silver, and other metals, including their oxides, have been recommended as antibacterial agents. The NPs are also engulfed by the mammalian cells through phagocytosis and are subsequently broken down through lysosomal fusions. The process effectively decreases the toxicity of the nanoparticles. Nonetheless, it causes free radical damage to the cells and the surrounding tissues. Thus, the metal nanoparticles possessed properties that enable them to inhibit the activity of bacteria responsible for infections. Also, the metal nanoparticles have pathways in the form of different mechanisms to harm the bacterial cells and cause them to be either dysfunctional or fatal to them. This mechanistic paraphernalia has bestowed the NPs with the ability to cause least, or nil damage to the mammalian cells, and has enabled the NPs, at the threshold of the size and exposure duration, to cause heavy toxicity which is not manageable. However, size-based toxicity of the NPs to mammalian systems and damage to tissues and organs at heavy and prolonged doses, and exposure time are documented^7^. The NPs can achieve greater bactericidal, and therapeutic effects than antibiotics and polymers because of their specific physicochemical characteristics and the molecular framework of their chemical structure^8,9^. The metal NPs have many of these properties of size, charge, and chemo-biological reactivity, that have made them particularly suitable for biological applications^10^. Among the prominent metal NPs, the gold NPs (AuNPs) exhibit a wide range of unique physico-chemical properties owing to their shape, naked and coated NPs reactivity, catalytic and oxidative behavior, surface charge, size and surface area, and higher ratio. The GNPs, in particular, have been well-accepted based on their demonstration of better biocompatibility, multifunctional nature, high stability, and water solubility^11^. The GNPs are known to hinder the growth of multidrug-resistant microorganisms by binding to the bacterial DNA and impeding the unwinding of DNA during the process of transcription^12^. This made the AuNPs (gold NPs) metal NPs of choice. The higher reactivity, size surface area, and reactivity control have been achieved through the NPs coating, and their embedding in another biocompatible and target-area suitable matrix. For this purpose, several materials, especially polymeric in nature have been used. Among the polymeric materials, biocompatible natural polymers have been on top of the list. Alginate, a natural polysaccharide polymer, obtained from marine algae, being a biodegradable, biocompatible, and nontoxic material has been found suitable for the purpose. The polysaccharide also works as the reducing material for the generation of nanoparticles and also provides suitability to the arising nanoformulation. The polymer’s present in the nanoformulation soup prevent their (the forming NPs) aggregation by decreasing the nanomatrix’s affinity and surface reactivity to their surfaces while maintaining the high innate bacteriocidal activity^13,14^. The chemical reduction provides simplicity, high yield, and low cost of the preparation^15^.

The anaerobic bacteria, such as *Cutibacterium* species, which are commensal microbes, have become opportunistic, and have led to dermatological disorders, such as acne^16^. The anaerobic bacteria lack an oxygen metabolism cytochrome system and hence are unable to eliminate toxic oxygen metabolites. As a result, they grow and survive in the obligate absence of oxygen. In an oxygenated atmosphere, these bacterium may respond negatively, or possibly die out. Nonetheless, these are part of the normal flora of human skin^17,18^. Skin anaerobic microflora, *e.g*. *Peptoniphilus asaccharolyticus, Anaerococcus prevotii*, and *Lactobacillus plantarum* have been sourced from different skin diseases^19–21^. Among other bacterial presence, the *Clostridium innocuum* is an intestinal bacterium. Recent studies have shown that the gut microbiome plays a role in acne, through interactions with the skin microbiome^22,23^. In the present study, gold/alginate nanoparticles (GANPs) was synthesized, and tested for their antibacterial activity against anaerobic bacterial strains isolated from acne patients. Furthermore, the hemolytic activity of the nanopreparation and its toxic effects on human white and red blood cells, and platelets has been determined.

## Materials and methods

### Chemicals and reagents

Chloroauric acid (HAuCl_4_.3H_2_O), sodium alginate, and crystal violet were purchased from Sigma Aldrich Chemical Co. St. Louis, MO, USA. Brain heart infusion media (BHI), and nutrient agar medium were provided by HiMedia, India. Anaerogen sachets were provided by Thermo Fischer Scientific, USA. All the chemicals and reagents were of analytical grade.

### Isolation of anaerobic bacteria from acne

**Figure 1:**
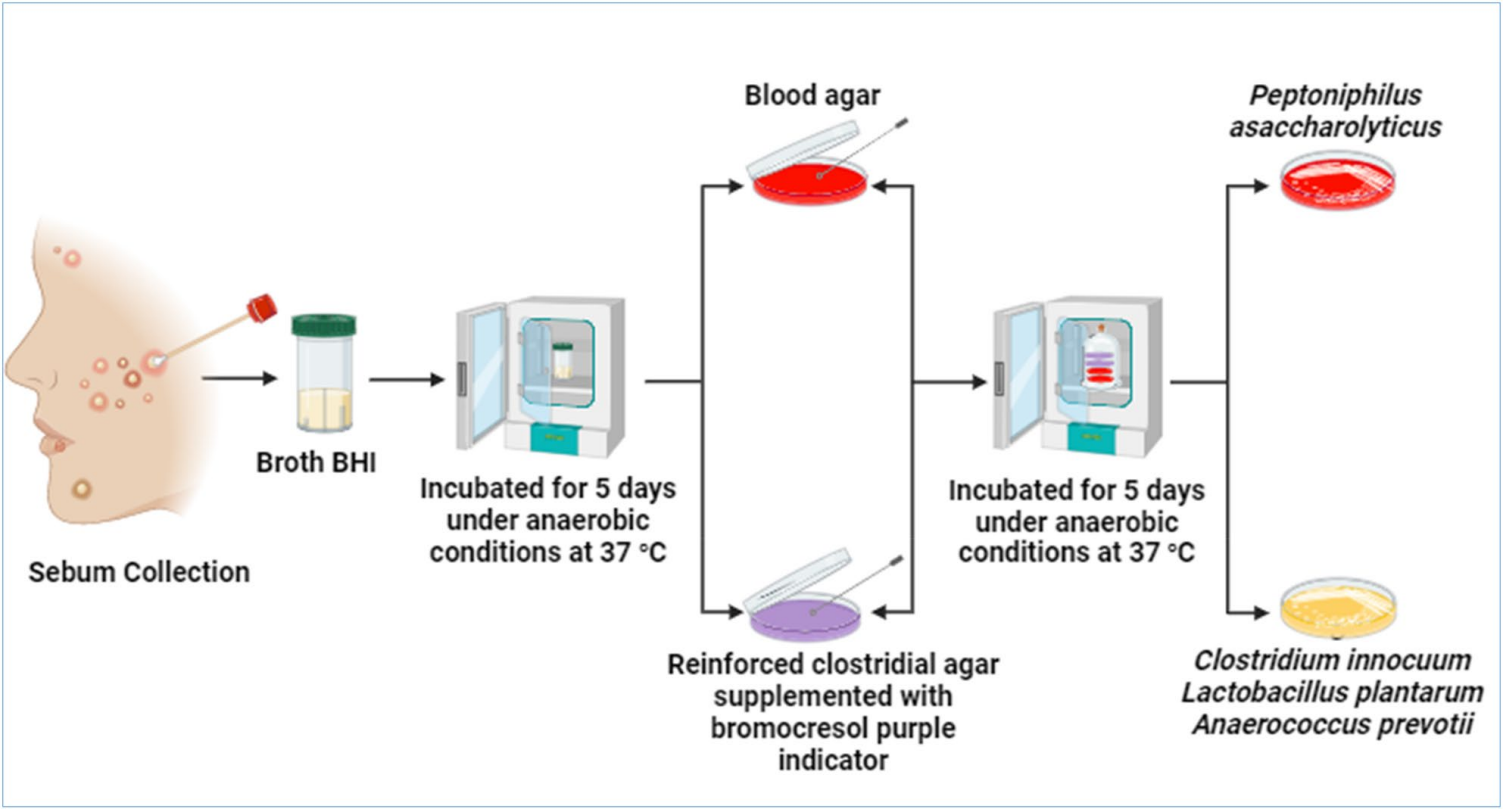
Isolation and identification of anaerobic bacteria.

A total of 70 acne patients, suffering from mild, moderate, and severe acne in their different regions, i.e., jaw, forehead, temple, chin, and cheeks, were enrolled in this study. All patients were from different places in the country. Requisite permissions were obtained from the hospitals and the study was conducted under Reference No. 4712 ASBT 8/11/2022 of the institutional approval. All participants agreed and signed written informed consent. The divisions of acne cases were divided into categories as (0–5) mild, (6–20) moderate, and (21–50) severe^24^. Gender, age, and distribution of acne on the face regions were considered. An alcohol pad was applied to the acne lesion to remove microorganisms found on top. Afterwards, the acne lesion was extracted a scratching of the tiny thin lancet, and the sebum was collected by gentle pressure with hand. The swab was placed in a screw cap tube containing 5 ml BHI broth as a transport medium. The tubes were incubated for five days at 37 °C in an anaerobic jar supplied with an anerogen sachet to create an anaerobic condition^25^, as represented in Fig. 1.

### Identification of bacterial isolates

According to the manufacturer’s protocol, culturing media, blood agar medium, and reinforced agar were prepared. RCA supplemented with bromocresol purple as an indicator (40 mg/L)^25^ was used. The swabs were anaerobically cultivated at 37 °C, for 5 days. The process was repeated twice for the colony's purification. The isolates *Clostridium innocuum*, *Lactobacillus plantarum*, *Peptoniphilus asaccharolyticus,* and *Anaerococcus prevotii* were identified based on microscopic examination and later confirmed with the VITEK 2 system (VITEK, Biomérieux, Marcy-l’Etoile, France), as represented in Fig. 1.

### Preparation of GANPs

**Figure 2:**
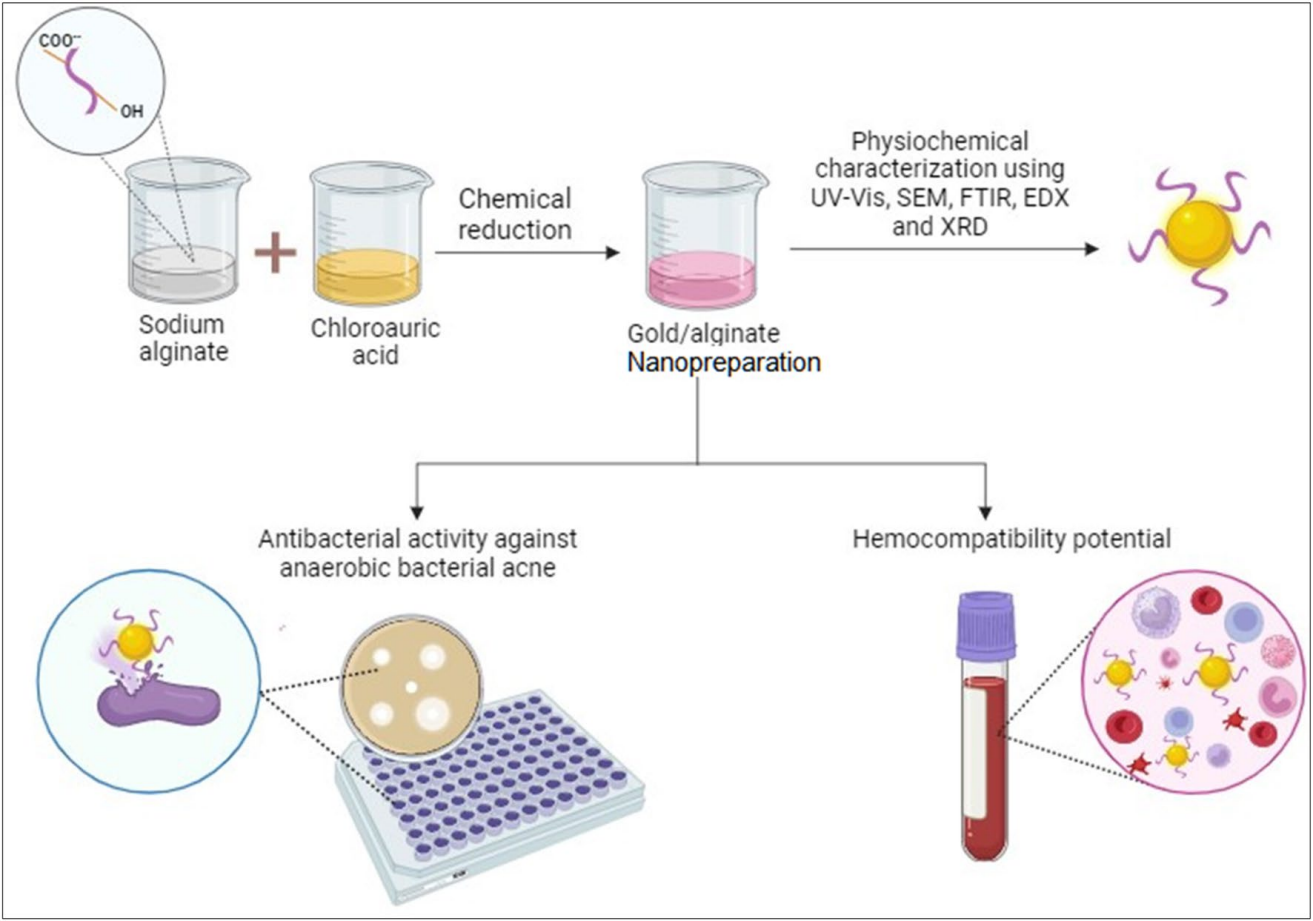
Illustration of GANPs preparation and main experiments.

Sodium alginate (1 g, 1%) MW_avg_ 12–40 KD was dissolved in 100 ml distilled water (DW). Chloroauric acid, HAuCl_4_, (67.9 mg) was dissolved in 10 ml distilled water (DW) to obtain 0.2 mM HAuCl_4_ solution. The dissolved chloroauric acid was added drop-wise to 5 mL of sodium alginate solution, and the mixture was heated to 80 ^ο^C for 40 min to facilitate the reduction of gold ions to provide the nanopreparation containing gold core and coated with the alginate polymer^26^. The prepared GANPs were used for further experiments as presented in Fig. 2.

### Characterization of GANPs

The absorbance values of GANPs, HAuCl_4_, and sodium alginate were determined by a UV–Vis spectrophotometer (UV–Vis) instrument purchased from Shimadzu 1900I, Japan. 5 ml of each sample was placed in a cuvette and measured with an absorbance range of 200–600 nm. The size and shape of each sample were studied using Scanning Electron Microscopy (SEM) through f50-FEI, Netherlands. Thin films of three different samples were fixed on a glass slide metalized for non-conductive samples, and examined under SEM. Energy dispersive X-ray spectroscopy (EDX; Axia, Netherland) was used to examine the chemical composition, while X-ray diffraction analysis (XRD; Aeris, Netherland) was used to determine the crystalline size, lattice parameter, and crystal’s atomic arrangements of each sample. All samples were analyzed by Fourier-Transform-Infrared Spectrophotometer (FTIR; Perkin Elmer, USA) within a wavelength range of 500–4000 nm to characterize the present functional groups in the samples.

### Determination of antibacterial activity of GANPs

#### Agar well diffusion method

The antibacterial effect of GANPs was assessed using the agar well diffusion method against strains of the isolated acne-sourced bacterial strains, i.e., *Clostridium innocuum*, *Lactobacillus plantarum*, *Peptoniphilus asaccharolyticus,* and *Anaerococcus prevotii*. The BHI agar plate was inoculated by spreading bacterial suspension over the agar surface. A well with a diameter of 6 mm was punched aseptically with a sterile cork borer, and GANPs 80 µl was loaded at different concentrations (6.4, 3.2, 1.6, and 0.8 mg/ml) into each well. DW was used as a controlling factor. The agar plates were incubated under anaerobic conditions at 37 °C for 48 h, and the results were obtained by measuring the inhibition zones by digital Vernier Caliper, USA. For each treatment, three duplicates were performed^27^.

#### Minimum inhibitory concentration (MIC)

A sterile microplate, 96-wells, was carried out to investigate the minimum inhibitory concentration (MIC) for GANPs. Each well contained 100 µl of BHI with a two-fold dilution of 6.4 mg/ml of GANP. Then, 100 µl of bacterial suspension, 10^8^ CFU/ml^28^, was gently mixed in each well, and incubated at 37 °C for 48 h in the anaerobic jar. The negative control contained 200 µl of nutrient broth, and the positive control contained 100 µl of bacterial suspension and 100 µl of free medium. Antibacterial activity was confirmed by adding 20 µL of resazurin to each well and confirmed through colorimetric observations of the samples. MIC was defined as the lowest concentration that inhibited bacterial growth. All experiments were carried out in duplicate^29^.

#### Anti-biofilm formation

Different concentrations of (100, 150, 200, 250, 300, 350, 400, 500, 600, and 700 µg/ml) of GANPs were dissolved in DW, and immediately 10 μl from each concentration were added to a single well in the microplate, which included 180 μl of BHI broth, and inoculated with 10 μl of bacterial suspension, 10^8^ CFU/ml. After incubation for 72 h under anaerobic conditions (37 °C), the contents of the plates were removed and washed 4 times with PBS (pH = 7) to remove free bacteria. Bacterial biofilms were fixed with 95% ethanol and stained with 0.1% (w/v) crystal violet. The plates were rinsed off 5 times with DW to remove excess stains and were kept dry. Following this, 225 µl of 33% glacial acetic acid was added, and readings were taken after 15 min by the microplate reader at 590 nm. The absorbance was considered the value of bacterial adhesion on the surface of nanoparticles and biofilm formation. The average of triplicate readings of each concentration was calculated at^30^.

#### Anti-adhesion assay

The adhesion tests were carried out by incubation of 200 µl of bacterial suspensions, 10^8^ CFU /ml in 96 wells of polystyrene microplates covered with a thin film of GANPs at concentrations (100, 200, 600, 700 µg/ml) and incubated for 4 h at 37 °C. The slides were washed three times with DW to fix the adhered cells, and 200 µl of methanol was flooded over each slide for 15 min. Thereafter, the non-adherent cells were removed by DW washing, and 200 µl of crystal violet (1%, w/v) was added and left for 15 min. The stained slides were washed with tap water, and the bound stain was solubilized by 200 µl of (33%, w/v) glacial acetic acid. At 630 nm, the optical density (O.D.) of the solubilized stains was measured by an automated plate reader^31^.

### Toxicity of GANPs on human blood parameters

Statement of ethical Permission was obtained from the hospitals and the institutional ethics committee of the Department of Applied Sciences, University of Technology, Baghdad, approved the study and its protocol (Ref. No. 4712 ASBT 8/11/2022). The NIH and Helsinki Declaration protocols were followed. Fresh human blood samples were obtained from healthy volunteers, and placed in tubes containing the anticoagulant agent, heparin. Each ml of blood was mixed with GANP and incubated at 37 °C for 4 h. The blood pictures were obtained using a hematological auto analyzer, Medonic M51, Sweden. To determine different hematological parameters, i.e., white blood cells (WBCs), lymphocytes (%), monocytes (%), neutrophils (%), eosinophils (%), basophils (%), red blood cells (RBCs), hemoglobin (Hb), hematocrit (HCT), and platelets (PLTs) were examined.

### Statistical analysis

ANOVA was used to analyze the data with the SPSS statistical program version IBM SPSS 29 (SPSS Inc., Chicago, IL). The mean and SD of three separate experiments were used to calculate the values.

## Result and discussion

### Characterization of GANPs

#### UV–Vis spectrophotometric analyses

**Figure 3:**
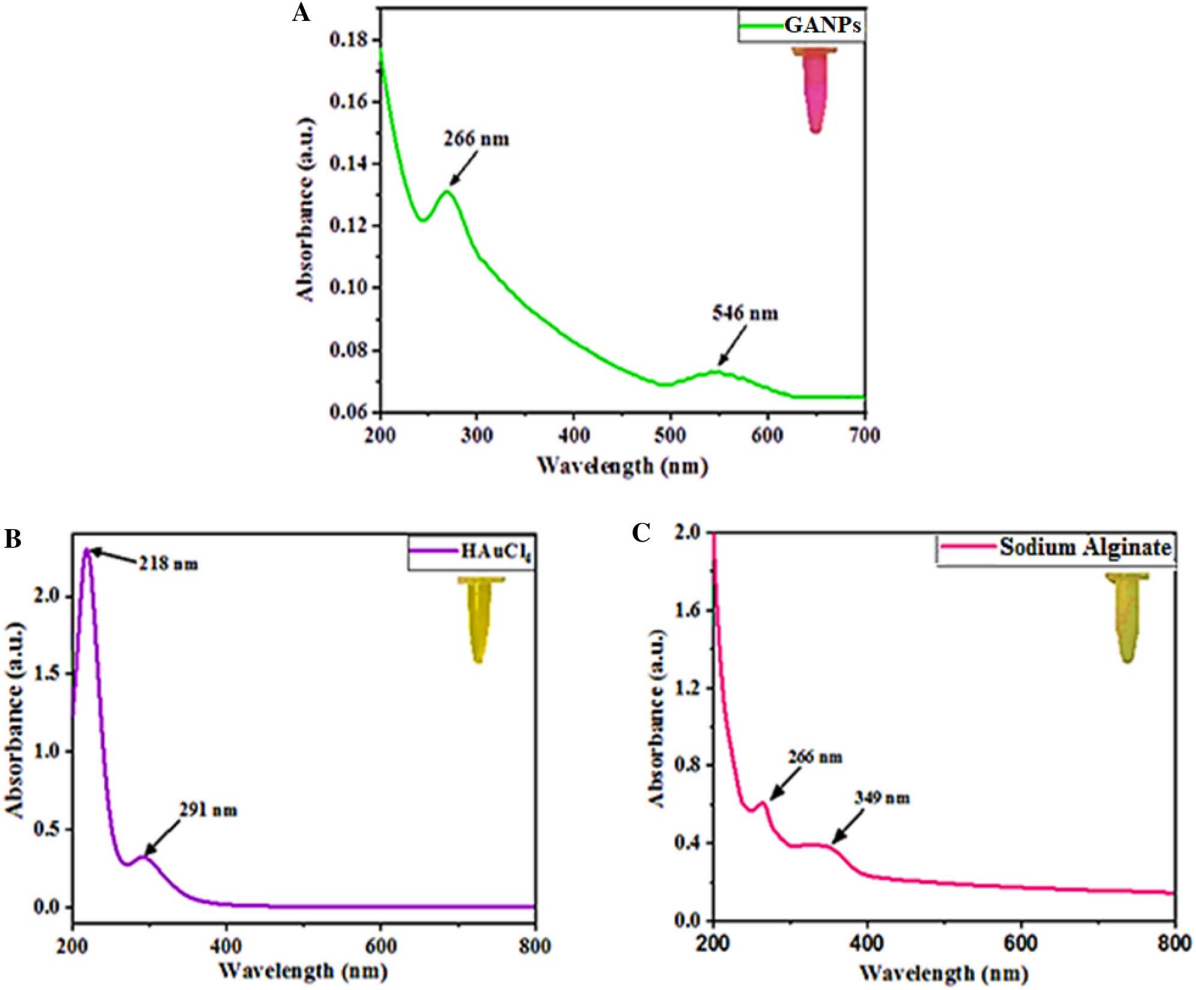
UV–Vis spectra of GANPs, HAuCl_4_, and sodium alginate.

UV–Vis spectrophotometric analyses of GANPs, HAuCl_4_, and sodium alginate solutions are presented in Fig. 3. The absorption maxima (λ_max_) of HAuCl_4_ were observed at 218 and 291 nm, which were assigned to the ligand–metal absorption owing to the charge transfer energies^32^. The λ_max_ for sodium alginate was observed at 266 and 349 nm. The reduction of HAuCl_4_ using sodium alginate as a reducing and stabilizing agent was confirmed principally by surface plasmon excitation causing the color shift from yellow to pink in the solution, which was the most substantial and apparent sign of the formation of GANPs. The surface plasmon vibration bands of nanoparticles (noble metals, particularly gold and silver) are much stronger than any other metals. The absorption spectrum showed the λ_max_ at 546 nm which was associated with the localized surface plasmon resonance (SPR) that confirmed the successful reduction of Au^3+^ into Au^0^ by the sodium alginate substrate^33^. The shift of the λ_max_ absorption band to a lower wavelength, as compared to the starting material, confirmed the reduction of the GANPs^34^. These results unequivocally indicated the effectiveness of the sodium alginate as a reducing, capping, and stabilizing agent wherein the product λ_max_ absorption band was observed at 546 nm as the newly appearing peak, while the presence of the λ_max_ absorption peak at 266 nm indicated the alginate presence, thereby confirming the alginate moieties presence on the gold nanoparticles in the nanopreparation. These findings also aligned with the previous report^26^.

#### Fourier transforms infrared (FT-IR) spectroscopic analyses

**Figure 4:**
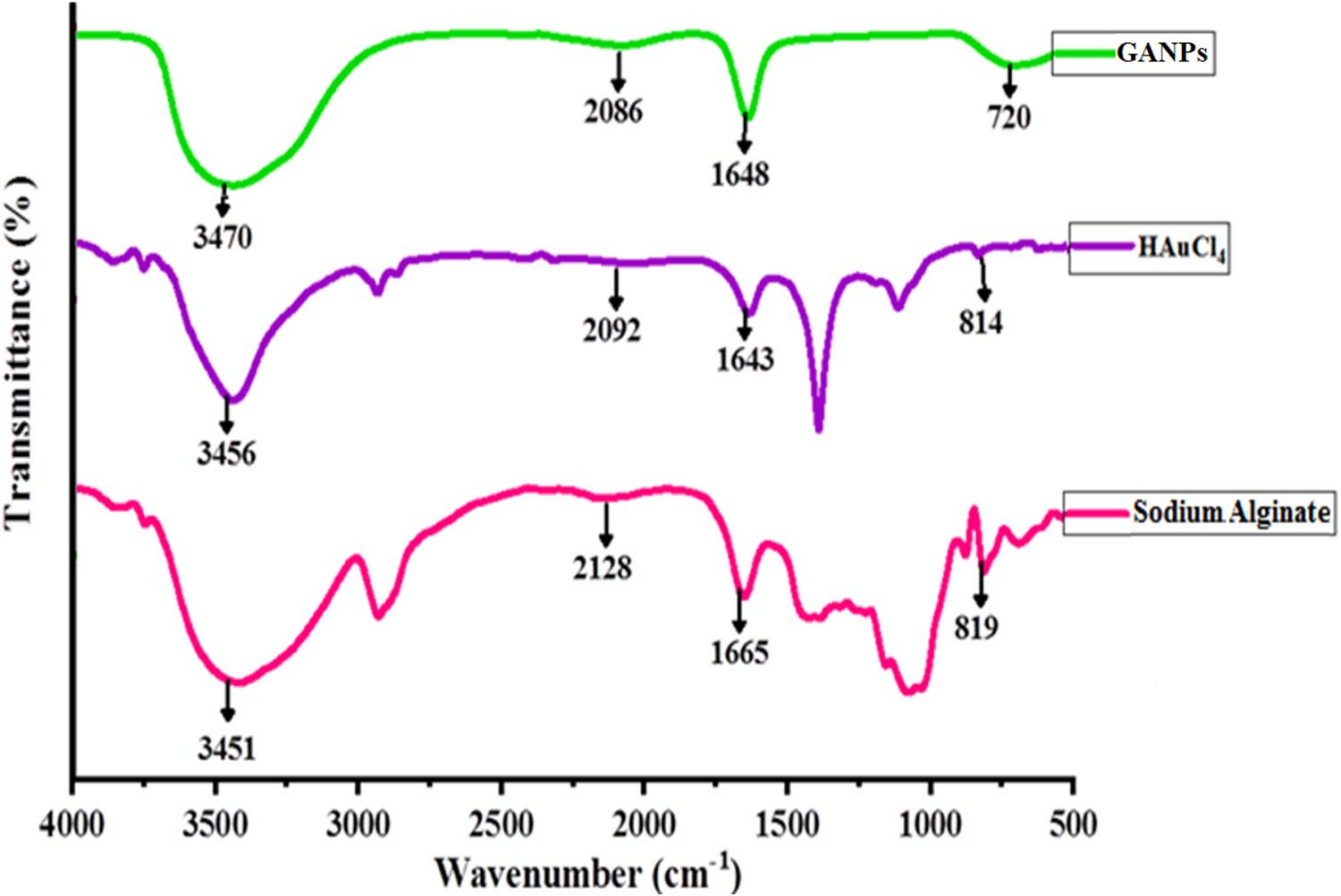
FT-IR spectrophotometric analysis of GANPs, HAuCl_4_, and sodium alginate.

FT-IR spectroscopy was employed to analyze the functional groups' presence in the GANP, HAuCl_4_, and sodium alginate. The comparisons of IR absorption bands of the starting materials and the prepared nano-product confirmed the formation of the intended nanopreparation (Fig. 4 and Table 1). Sodium alginate showed absorption peaks at 3451, 2128, 1665, 1426, 1060, and 819 cm^–1^^35^. The absorption peak at 3451 cm^–1^ was assigned to the free hydroxyl groups, while the peaks at 1665 cm^–1^ were allotted to the carbonyl functionality. For the HAuCl_4_, several absorption peaks at 3456, 2092, 1643, 1393, 1123, and 814 cm^–1^ were observed. The spectrum of GANPs showed four different peaks at near similar wavelengths in comparison with the sodium alginate and HAuCl_4_. The four peaks of the GANP were observed at 3470, 2086, 1648, and 720 cm^−1^, respectively. The peak at 3470 cm^−1^, was assigned to the O–H stretching vibration, while peaks at 2086 cm^–1^ and 1648 cm^−1^ corresponded to the asymmetric stretchings of C=C and C=O groups^36^. The peak at 720 cm^−1^ was referred to as the alkane functionalities of the GANP. The O–H groups peak shift from 3456 to 3470 cm^–1^, indicating the interactions between the alginate moiety and the HAuCl_4_ starting materials wherein the redox between the alginate and the gold substrate exchanged electrons to reduce the Au^3+^ ions to Au^0^ through the reduction process^37,38^.

**Table 1:**
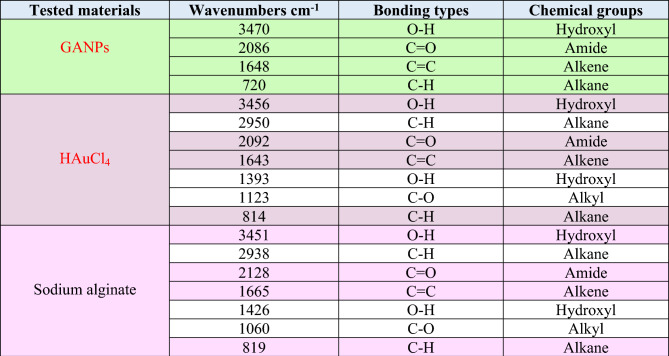
FTIR bonding types and chemical groups of GANPs, HAuCl_4_, and sodium alginate.

#### Scanning electron microscopic analysis

**Figure 5:**
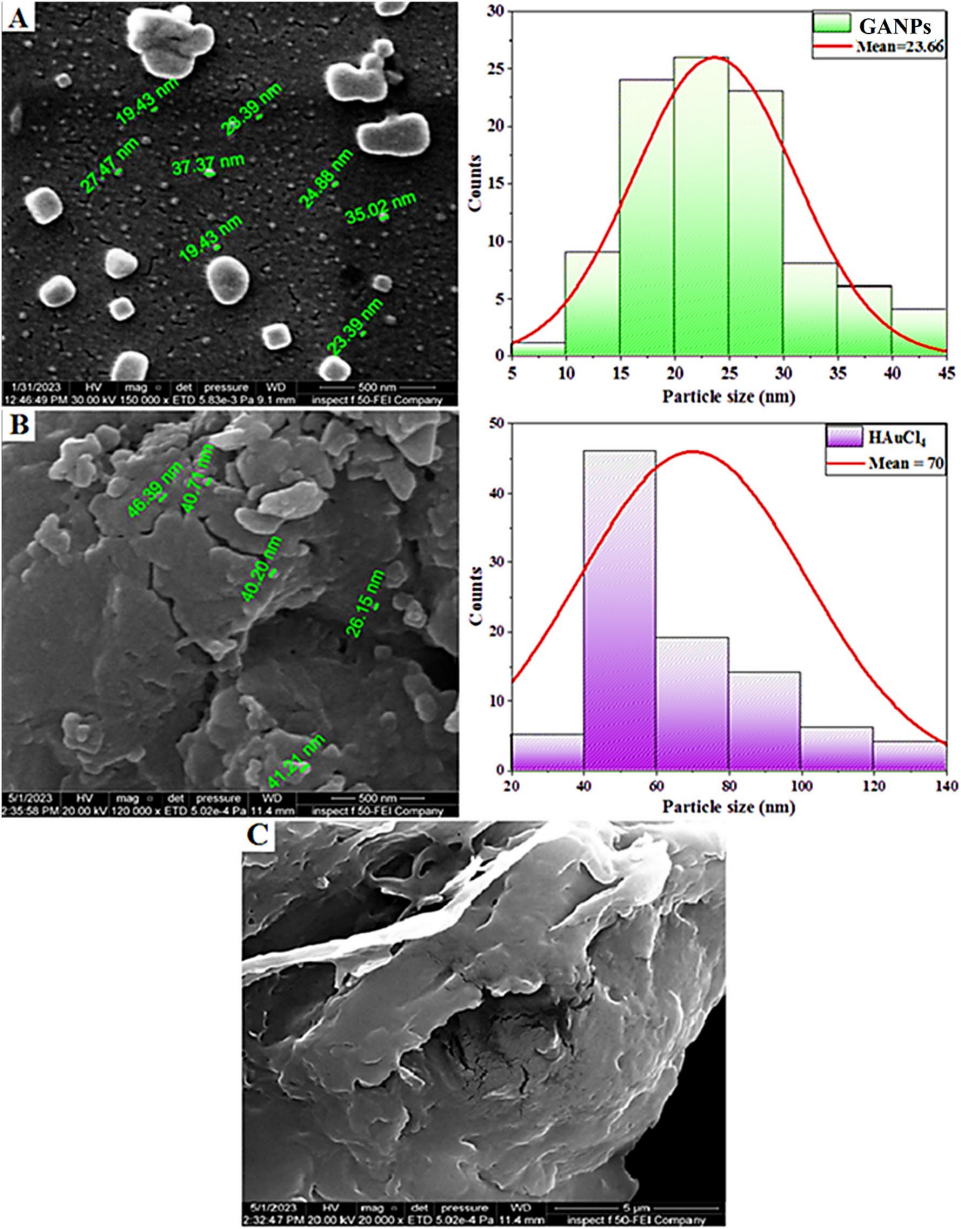
SEM analyses of (**A**) GANPs, (**B**) HAuCl_4_, and (**C**) sodium alginate.

Figure 5 represents the SEM magnified pictures of the starting materials and the final product. The surface morphology of the GANPs (magnification 150,000×) confirmed the formation of nanoparticles, which are present in the GANP. It showed no aggregation and smooth surface of the nanoparticles^26^ (Fig. 5A). Figure 4B,C showed the magnified pictures of the raw materials, HAuCl_4_, and sodium alginate. The sodium alginate showed a highly porous and rough surface, that was feasibly conjugated with the forming AuNPs during the process of preparation of the nano-product in the aqueous environment, thereby also providing it coating/capping and stabilization^39^. The SEM analysis of the HAuCl_4_ (120,000$\times$) showed comparatively bigger sizes, and irregular shapes with a mean particle size of 70 nm which was expected due to the HAuCl_4_ clusters and its spherical morphology (Fig. 5B). The alginate analysis is shown in Fig. 4C. The histogram analyses showed a mean size of 23.66 nm of the prepared nanoparticles. The findings are consistent with previous report^40^.

#### Energy dispersive X-ray analysis

**Figure 6:**
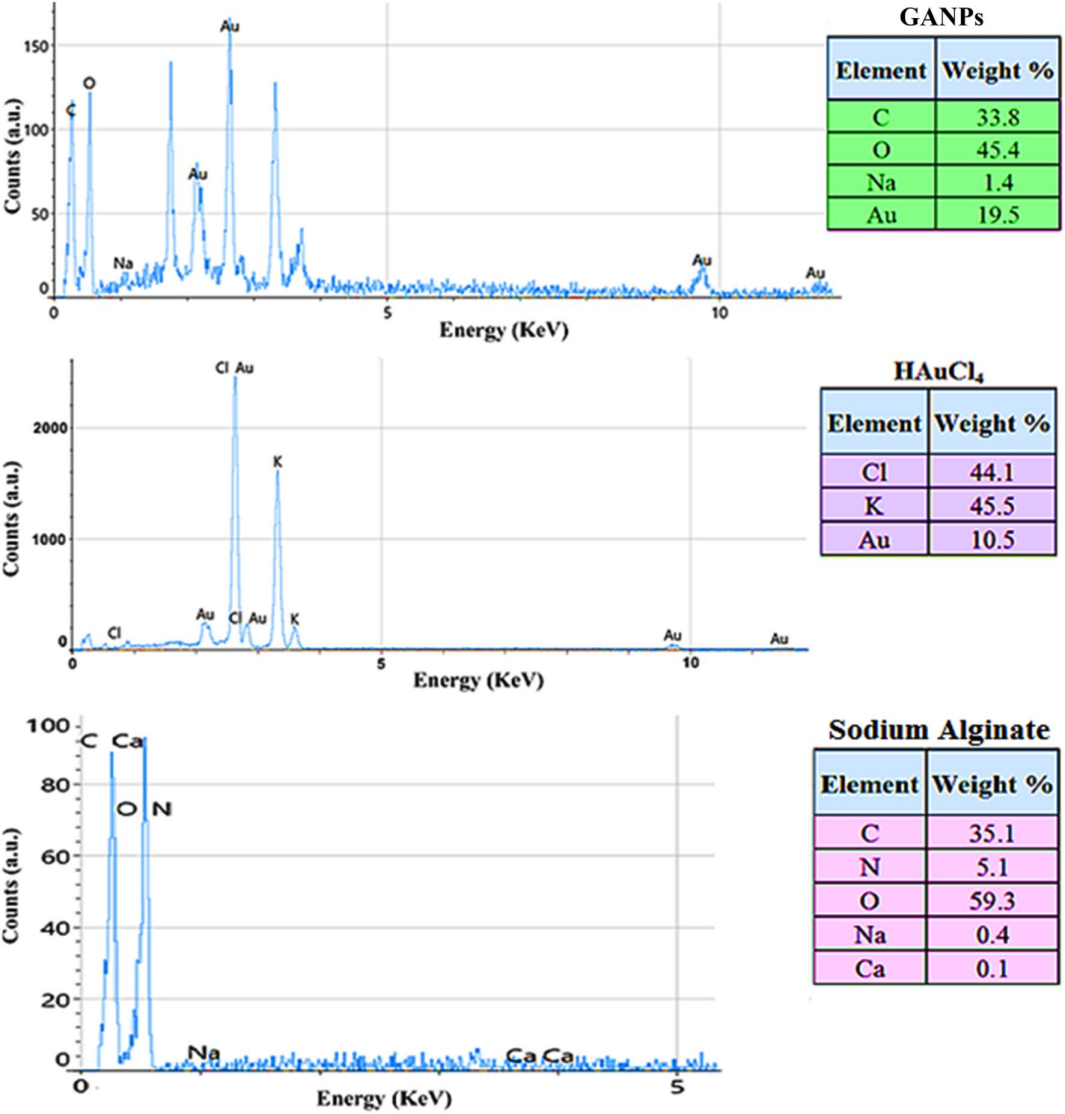
EDX spectroscopy of GANPs, HAuCl_4_, and sodium alginate.

The EDX analysis showed the elemental peaks of the carbon (C), nitrogen (N), oxygen (O), sodium (Na), and calcium (Ca) atoms at the energy value of 5 keV for the sodium alginate. The elemental peaks of chlorine (Cl), potassium (K), and gold (Au) were also observed at the 5 keV energy values in the EDX spectra of the HAuCl_4_. The E-mapping analysis of the GANP confirmed the presence of Au, Na, O, and C in the sample of prepared final nanomaterial which demonstrated the characteristic peak of Au with a weight percentage of 19.55, while the percentages of O and C were 45.4 and 33.8, respectively. The C is sourced from the alginate moieties that are present in the nanoparticles of the prepared nanomaterial. These findings are consistent with the previous report^41^ (Fig. 6).

#### X-ray diffraction (XRD) analysis

**Figure 7:**
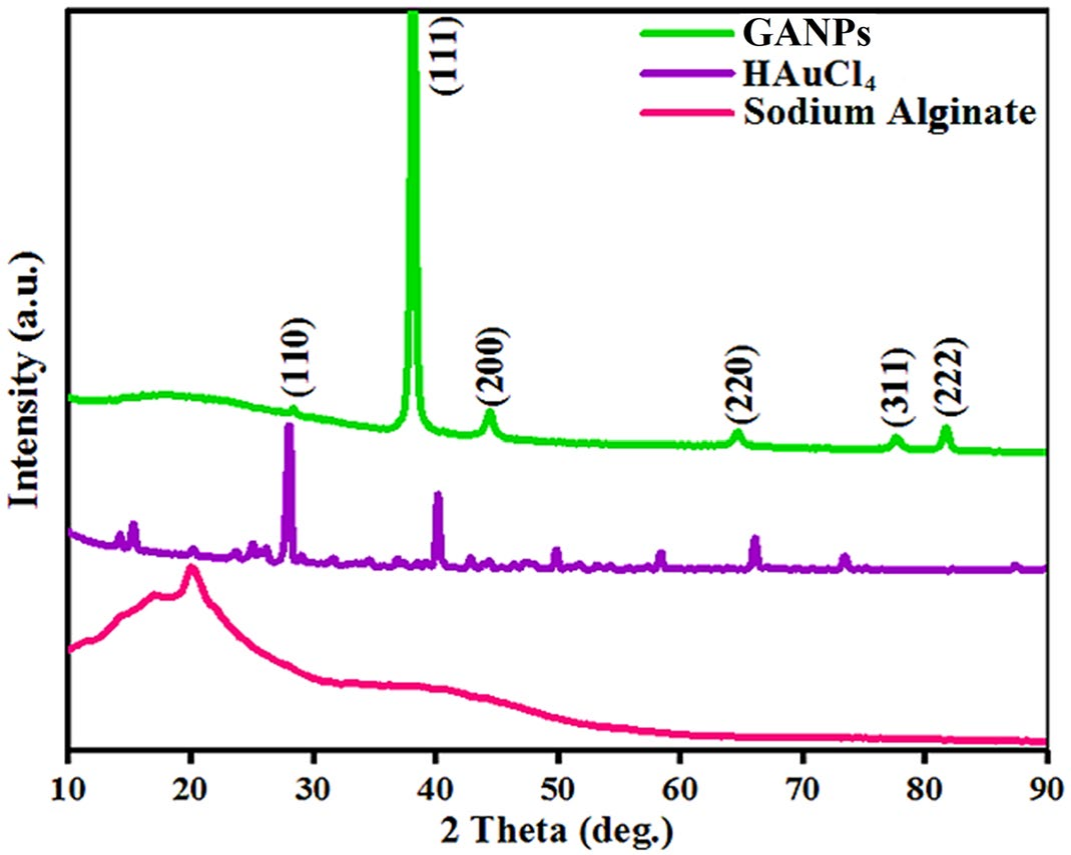
The XRD analyses of GANPs, HAuCl_4_, and sodium alginate.

The XRD of GANPs (Fig. 7) at 2Ө orientation showed six peaks at 28.68°, 38.18°, 44.39°, 64.57°, 77.54°, and 81.72°. These peaks corresponded to the (110), (111), (200), (220), (311), and (222) planes, respectively, when compared with the Joint Committee on Powder Standards (JCPDS), File no. 04-0784 and 01-074-0895. These peaks confirmed the crystalline nature of the GANPs and provided confirmatory proof that the material can be indexed as a face-centered-cubic structure^42,43^. Out of the related plane, the (111) plane showed a higher intensity than other planes, demonstrating that the (111) plane is the typical orientation. In case of HAuCl_4_, six peaks, 15.36°, 28.113°, 40.366°, 50.317°, 58.316°, and 66.148° were detected at angle 2Ө which corresponded to planes (200), (− 112), (021), (− 421), (− 711), and (− 803), respectively, as compared with the JCPDS, File no. 01-073-1234. The XRD of sodium alginate at 2Ө observed two peaks at 19.84° and 28.68° due to the reflection of their (001) plane from the amorphous halo and (110) plane from the polyguluronate unit, the findings are consistent with the previous report^44,45^. The lattice size (D) of GANPs was also collected using Scherer’s equation which predicted the average size of the nanoparticles of the prepared nanomaterial GANPs at about 37.53 nm.

### Isolation and identification of anaerobic bacteria from acne

**Figure 8:**
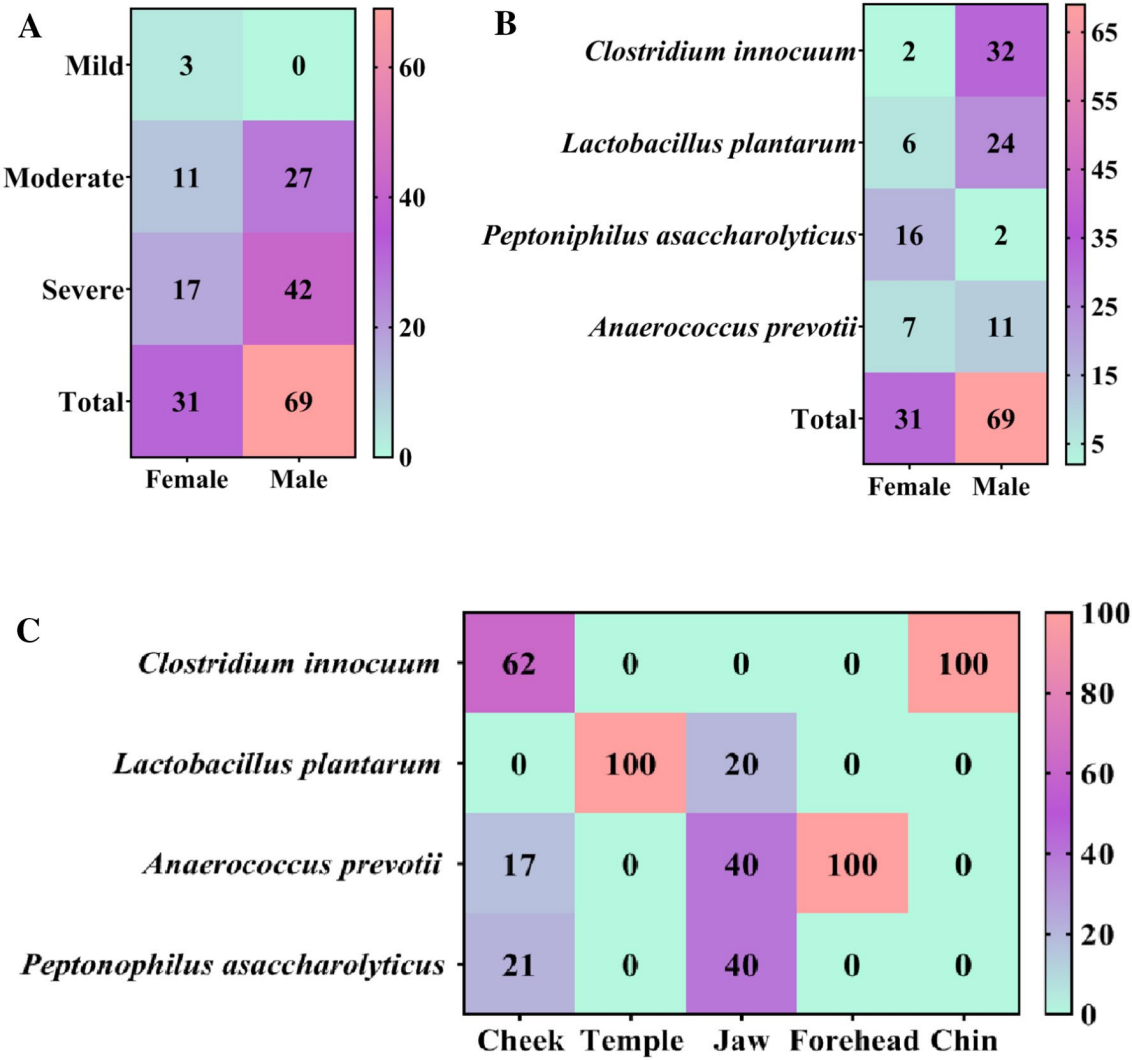
Percentage distribution of acne: (**A**) gender-wise distribution and severity of acne, (**B**) gender-wise distribution of bacterial acne, and (**C**) relative bacterial abundance with face regions.

The study included 70 patients with acne vulgaris. The male patients' percentage was 69% while females constituted 31% (Fig. 8A). This is because men delay treatment, and are more exposed to the factors of the outside environment, such as exposure to the sun, high temperature, air pollution, halogenated hydrocarbons use, inhaled and passive exposures, as well as exposures to mineral oils, all that serve as risk factors for acne. Also, in personal hygiene-related habits wherein facial cleansers for the skin are consistently used, certain studies have indicated a significant relationship between the severity of acne and personal hygiene^46^. Acne can be affected by factors, *e.g*., family history, the nature of the skin, oily and mixed skin, obesity, sweetened food, smoking, poor quality of sleep, and stress^47^. Also, the usage of skincare products, especially in females, the acne incidence was statistically higher in females at 74.5%^48^. Figure 8B summarizes the distribution of acne, gender, and regions of acne together with acne-sourced bacterial strains between the genders. Herein, the males showed the highest infection by *Clostridium innocuum*, which may be because of smoking, fast food, and hygienic causes, as it is among the most common bacterial infections that could translocate from body part to the skin and tissues and cause serious effects^49^. The age of acne patients was between 15 and 30 years, which could be due to the enlargement of keratinocytes and sebaceous glands over-activity resulting in excessive production of oily sebum causing acne. Moreover, other factors, such as school-age stress also result in the prevalence of acne^50^. Finally, the results indicated that different acne sampling sites have different types and numbers of acne-sourced bacterial strains. It was observed that among the face sampling sites, the cheek and jaws were mostly containing the anaerobic bacterial types. Any alteration of the skin bacteria composition changes the functions of skin microbiota and affects the development and occurrence of skin disease. It is important to mention that most of these functions are associated with the metabolism of essential chemicals, such as lipids, cofactors, vitamins, and amino acids. The alteration in these compounds may also significantly disturb the host skin homeostasis through stimulation of the immune cells and keratinocytes of the skin^51^ (Fig. 8C). The heat map showed the distribution of these bacteria on different face regions, and from these clinical specimens of bacteria, 24 were *C. innocuum*, 21 were *Lactobacillus plantarum*, 13 were *Anaerococcus prevotii*, and 12 were *Peptoniphilus asaccharolyticus* that were isolated to investigate the antibacterial activity potential of GANPs.

### Antibacterial activity

#### Agar well diffusion tests

**Figure 9:**
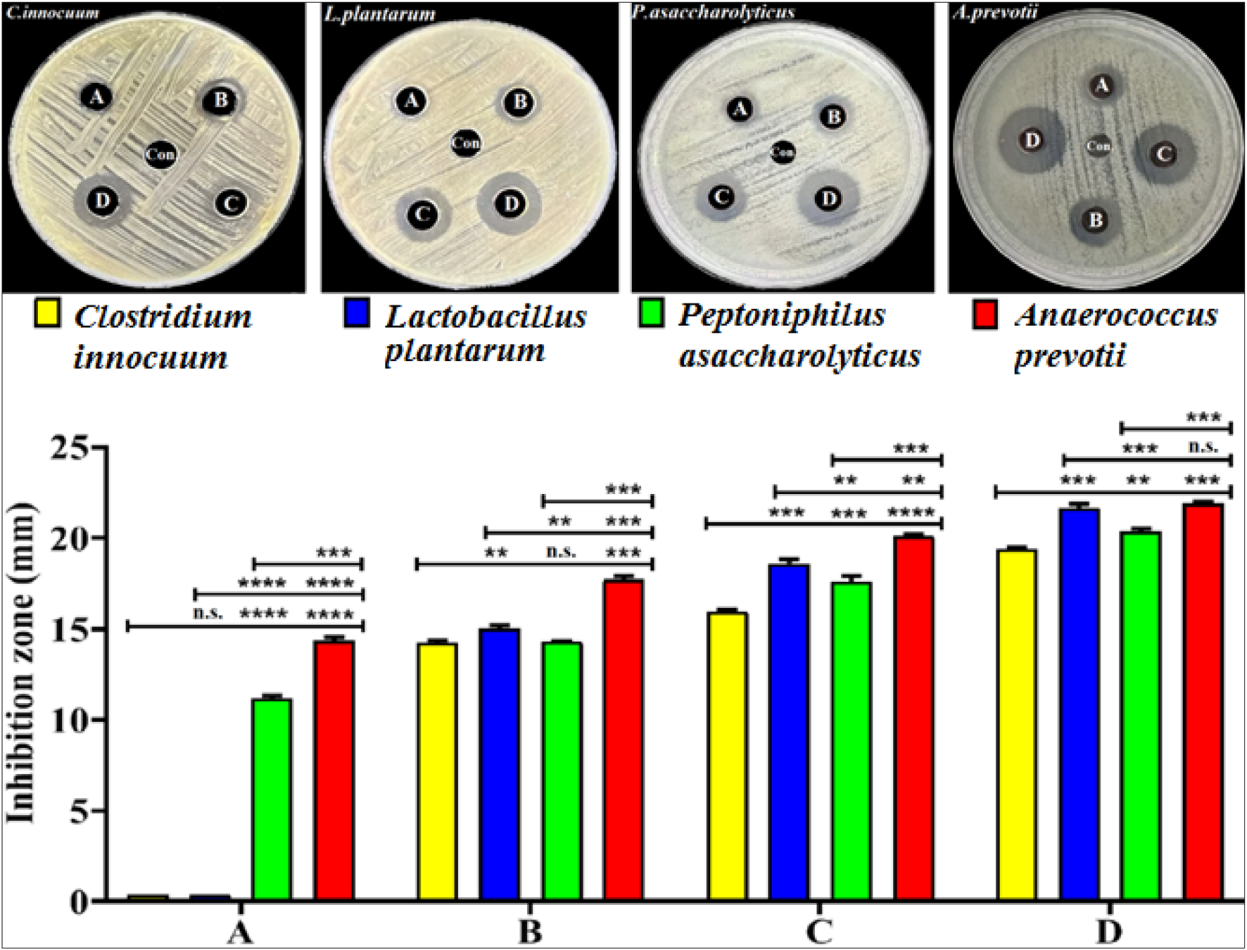
Inhibition zones by GANP against *C. innocuum*,* L. plantarum, P. asaccharolyticus*, and *A. prevotii* at concentrations of (**A**) 0.8, (**B**) 1.6, (**C**) 3.2, and (**D**) 6.4 mg/mL. The values are shown as the mean ± SD. from three replicate experiments. The n.s. refers to not significant, *p < 0.05, **p < 0.01, ***p < 0.001, ****p < 0.0001.

The antibacterial activity of GANPs at various concentrations (6.4, 3.2, 1.6, and 0.8 mg/ml), revealed the inhibitory effects of the nanopreparation against *C. innocuum*, *L. plantarum*, *P. asaccharolyticus,* and *A. prevotii *(Fig. 9). The bacteria with the highest inhibitory diameter in the agar well-diffusion tests were *A. prevotii*, with an average inhibition zone diameter of 21.88 mm at 6.4 mg/ml concentration, followed by *L. plantarum* with an average inhibition zone diameter of 21.68 mm, and *P. asaccharolyticus* with an average inhibition zone diameter of 20.43 mm, respectively. The bacteria with the lowest inhibitory diameter in this study was *C. innocuum* which showed an average inhibition zone diameter of 19.37 mm at 6.4 mg/ml, owing to their ability to produce toxin-forming spore-forming that made it more resistant to most of the antibiotics^22^. The Gram-positive bacteria were more resistant to the antimicrobial mechanisms of nanoparticles. The thickness of the peptidoglycan layer found in Gram-positive bacteria cell walls was thought to be responsible for this resistance^52^. The GANPs are regarded as antimicrobial agents able to generate a clearance zone for antimicrobial activity due to its nano-sized components and better penetration ability. The GANPs can bind to the cell membrane, disrupt the permeability of the outer cell membrane to penetrate the inner layer of the cell membrane, and block the respiratory chain dehydrogenase, together with disassociating the respiratory chain and oxidative phosphorylation, thereby also disabling the proton channel force through the cytoplasmic membrane. By reducing the size of the nanoparticles, the interaction between the nanoparticles and the bacterial cell may result in increased release of the cored gold particles to improve the antibacterial properties. The electrostatic attraction between the bacterial cell membrane and the GANPs may also lead to an increased accumulation of gold on the bacterial cell membrane, which can lead to high stress on the bacterial membrane and the further penetration of GANPs into the cytoplasm, eventually leading to cell lysis^53,54^. The current findings agreed with Li et al*.* who demonstrated that the AuNPs owing to their favorable surface chemistry, may be utilized as an antibacterial agent against multidrug-resistant Gram-positive and Gram-negative bacterial pathogens^55^.

#### MIC analysis

**Figure 10:**
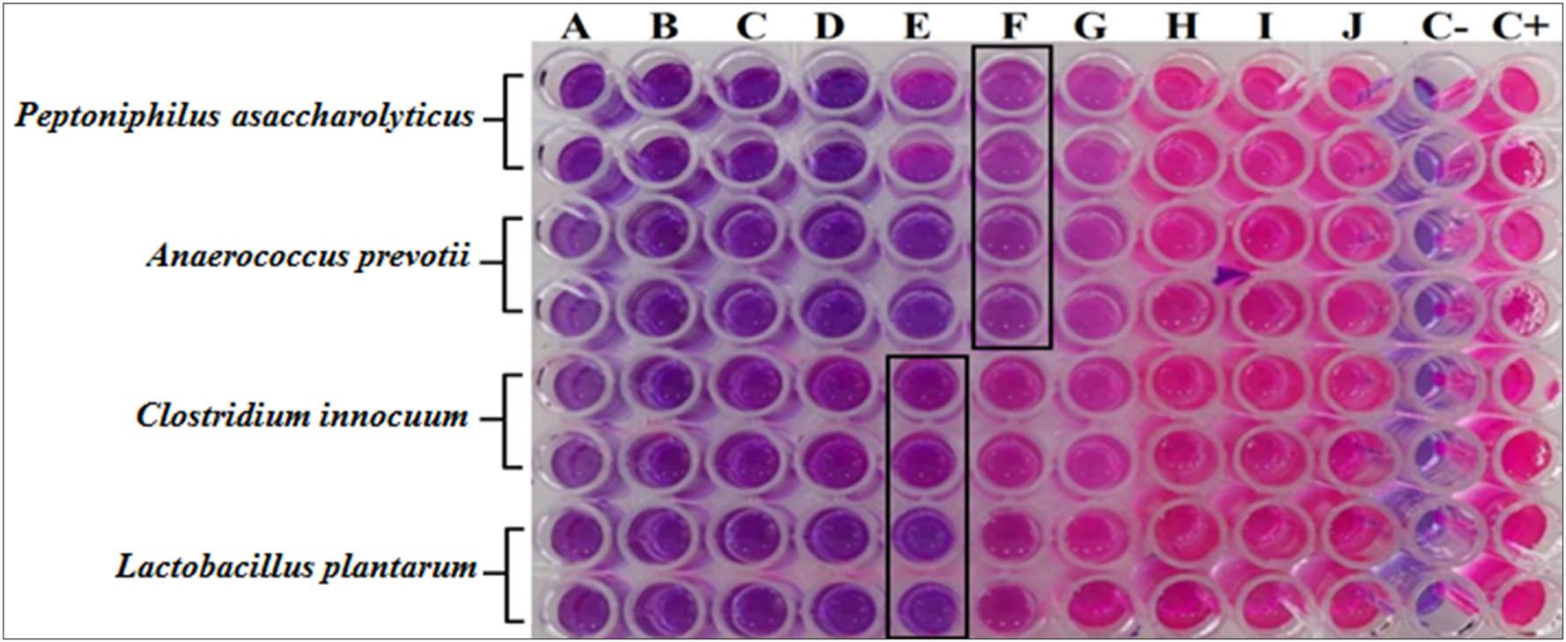
MICs determinations of GANPs against *Peptoniphilus asaccharolyticus*, *Anaerococcus prevotii, Clostridium innocuum*, and *Lactobacillus plantarum.*

The MIC of GANP at various concentrations was tested with 6.4 mg/ml to 12.5 µg/ml against *C. innocuum*, *L. plantarum*, *P.s asaccharolyticus,* and *A. prevotii*. The influence of GANPs on the growths of bacterial isolates from acne was determined colorimetrically using resazurin. The GANPs showed excellent potential against these pathogenic bacteria. From 6.4 mg/ml to the concentrations of 400 µg/ml, complete killing of the bacteria in the wells was recorded in the wells. The MIC of GANP for *P. asaccharolyticus* and *A. prevotii* at a concentration of 200 µg/ml showed a color change to pink due to the activity of bacteria, while the MIC for *C. innocuum* and *L. plantarum* was at 400 µg/ml. These results showed that *P. asaccharolyticus* and *A. prevotii* were more sensitive to GANP (Fig. 10). Seemingly, following the previous report, the GANP also exhibited antibacterial effects in two ways, first, they restricted the membrane potential, and interacted with the mitochondria to inhibit the ATPase and then reduce the ATP levels, indicating a general decline in metabolism. Second, affects bacterial DNA by preventing ribosomal subunits from attaching to tRNA. The GANP improved the chemotaxis in the early phases of the biochemical reactions. The mechanism of action of gold nanoparticles didn’t include pathways associated with reactive oxygen species (ROS), which causes cell death by bactericidal antibiotics^56^. The results from this study demonstrated the GANP’s significant anti-bacterial activity at lower concentrations against anaerobic bacteria sourced from the acne.

#### Anti-biofilm analysis

**Figure 11:**
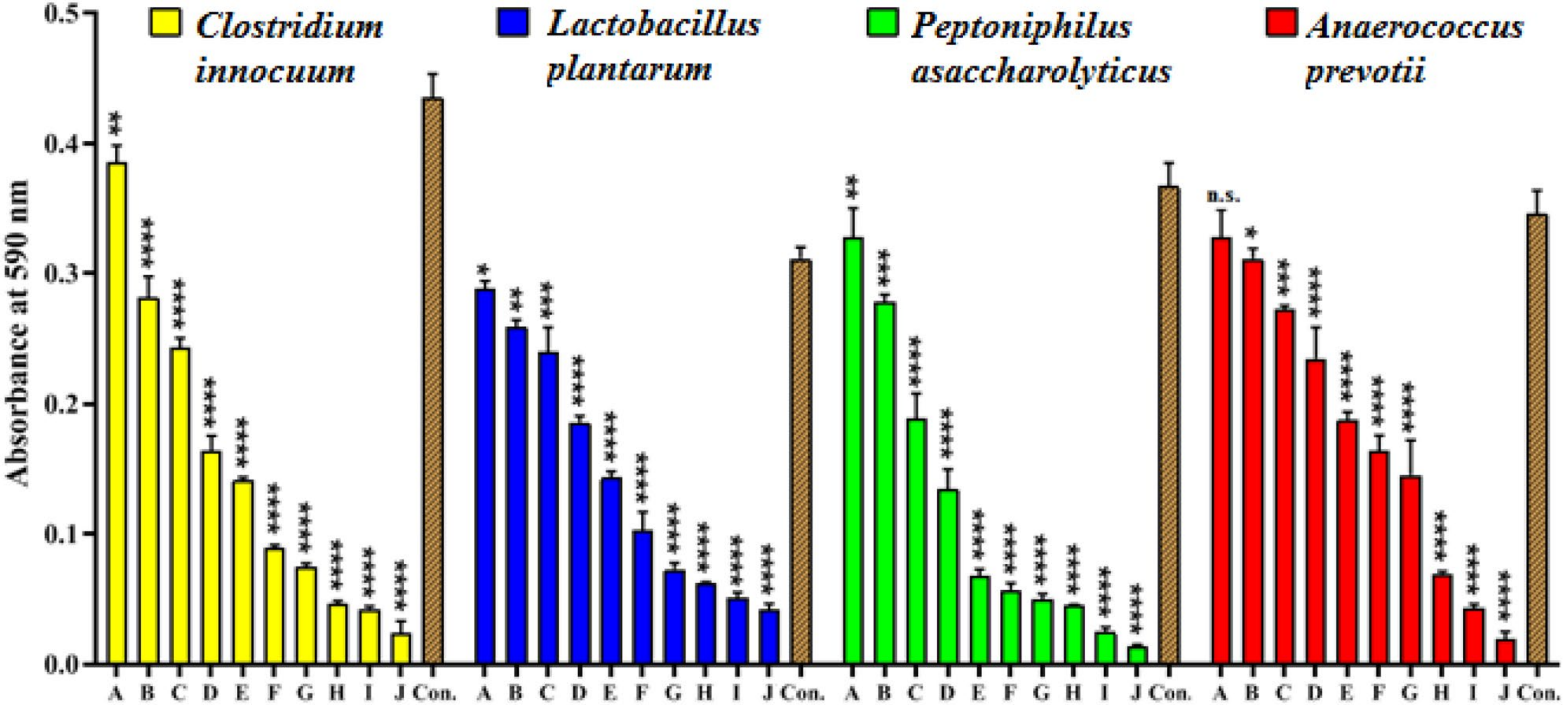
Anti-biofilm assay of GANPs at different concentrations on biofilm formation by *Clostridium innocuum*, *Lactobacillus plantarum, Peptoniphilus asaccharolyticus*, and *Anaerococcus prevotii.* with positive control, according to optical density values at 590 nm after incubation for 72 h. The values are shown as the mean ± SD. from three replicate experiments. The n.s. refers to not significant, *p < 0.05, **p < 0.01, ***p < 0.001, ****p < 0.0001.

The anti-biofilm activity of GANPs at various concentrations (100, 150, 200, 250, 300, 350, 400, 500, 600, and 700 µg/ml) against *C. innocuum*, *L. plantarum*, *P. asaccharolyticus,* and *A. prevotii* were evaluated utilizing a microtiter plate staining procedure with crystal violet and measurements by a microplate absorbance reader at 590 nm. The addition of GANPs inhibited the formation of biofilm of most of the acne-sourced bacterial strains, as indicated by a decrease in the optical density values with the rising concentrations of the nanopreparation (Fig. 11), The GANPs exhibited significant changes in all the bacterial strains from the highest up the concentration of 700 µg/ml to the lowest, except for *A. prevotii* which showed activity at 100 µg/ml, which did not prevent the formation of biofilm. Two different concepts have been proposed to explain these effects, the GANPs may weaken the biofilm formation in bacteria by moving into the bacterial cell wall and interfering with the enzymes and proteins required for microbial adhesion, which results in decreased biofilm formation^57^. The GANPs inhibits biofilm formation by inhibiting the formation of exogenous polysaccharides. The NPs inhibit the biofilm formation by penetrating the water channels /aqua pores that transport water and nutrients through the layers of polysaccharides present in the bacterial cell walls^58,59^. The ongoing results have demonstrated the impact of GANPs on the formation of bacterial biofilms and significant variations were noted with the change in the bacterial species, types of nanoparticles, nanoparticles’ size, and nanoparticle concentrations. These results agreed with Al-shukri et al. findings^60^ which demonstrated that AuNPs at high concentrations (75, 100, and 200 µg/ml) influenced the formation of biofilms as compared to the low concentrations of the AuNPs.

#### Bacterial anti-adhesion analysis

**Figure 12:**
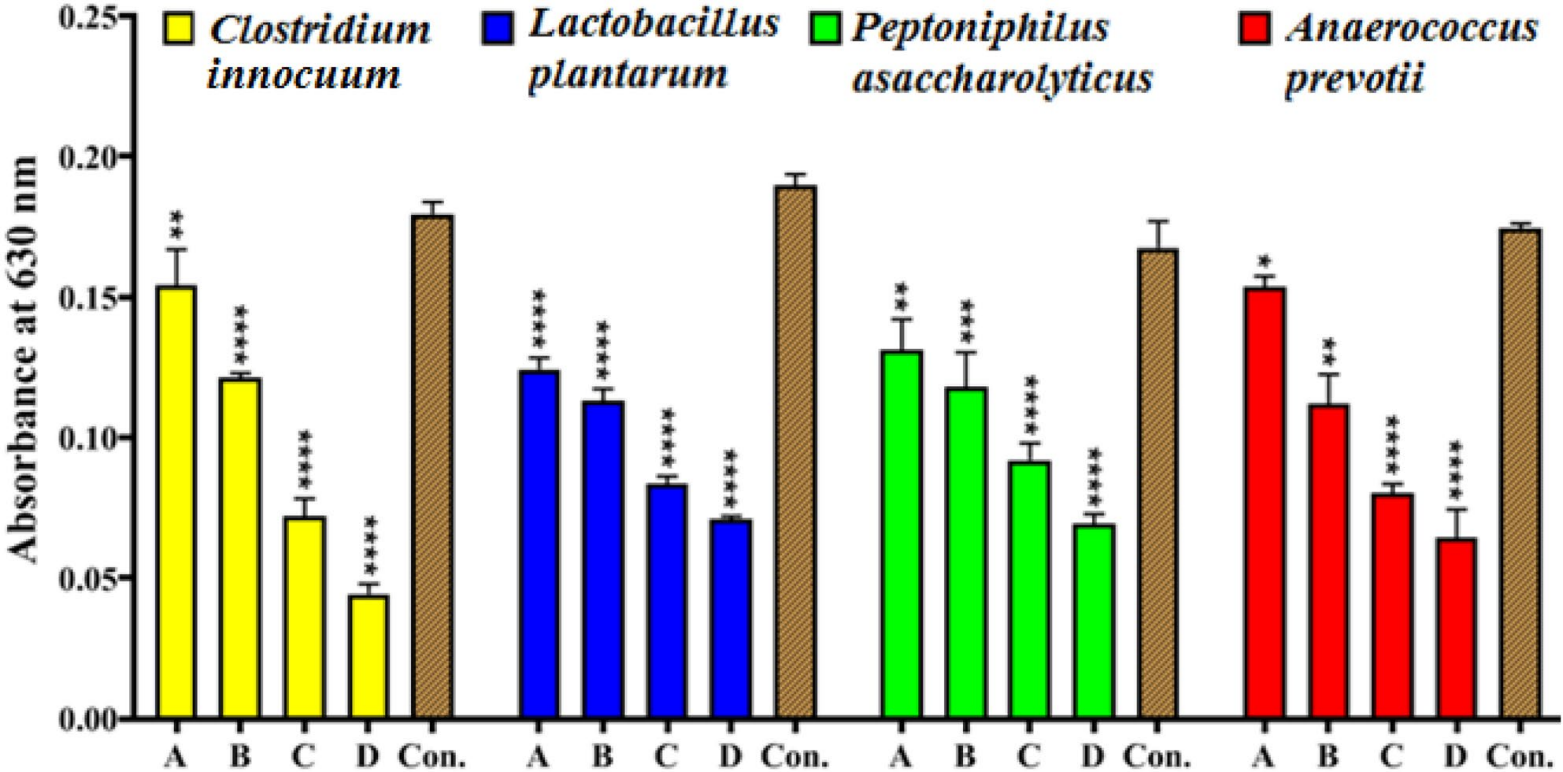
Anti-adhesion assays of GANP at different concentrations against *Clostridium innocuum*, *Lactobacillus plantarum, Peptoniphilus asaccharolyticus*, and *Anaerococcus prevotii* with positive control, according to optical density values at 630 nm after incubation for 4 h. The values are shown as the mean ± SD. from three replicate experiments. *p < 0.05, **p < 0.01, ***p < 0.001, ****p < 0.0001.

The anti-adhesion activity of GANPs at various concentrations (100, 200, 600, and 700 µg/ml) against *C. innocuum*, *L. plantarum*, *P. asaccharolyticus,* and *A. prevotii* were evaluated utilizing a microtiter plate staining procedure with crystal violet and measurements by a microplate absorbance reader absorbance investigation at 630 nm (Fig. 12). The addition of GANPs inhibited the initial adherence of acne-sourced bacterial strains to polystyrene microplate, as indicated by a decrease in the optical density (O.D.) values with the rising concentration of the GANP nanoformulation. The role of GANPs as an antimicrobial agents involved two steps upon incubation with bacterial cells. First, the aggregation of GANPs under bacterial cells followed by interaction with the surface after reaching it, causes an increase in cell wall tension. Secondly, after penetration, the nanomaterial-based nanoparticles migrate into the interior of the biofilm and bacterial cell components causing nano-toxicity leading to metabolic imbalance followed by bacterial cell death. The GANPs penetration depends on several factors, such as the structure of the biofilm surface, chemical composition, biofilm maturity, size of the nanoparticles, surface charge, and nanoparticle concentration. The antimicrobial potentials of GANP were dependent upon the perturbations that occurred in different bacterial components and the biofilm. All biofilm penetration methods involved hydrophobic interaction, hydrogen bonding, electrostatic, and van der Waals attraction-based interactions^61,62^. The current results were in agreement with previous result^63^ wherein it was exhibited that the AuNPs capping by Arabic Gum exhibited a noteworthy anti-adhesion activity against *Cutibacterim* acne.

### Effect of GANPs on human blood components

The human blood components were treated with 1 mg/ml of GANPs and exhibited no significant effects as compared with the control (Table 2). The hemolysis percentage of the RBCs was at 1.27% which was the acceptable level of less than 5% standard, under limited hemolysis. Following the criterion in the ASTM E2524-08 standard (test procedure for analyzing the hemolytic properties of nanoparticles), greater than 5% hemolysis suggests that the test nanoparticle damaged the RBCs^64^. A quantitative analysis of the released hemoglobin also indicated potential damage of RBCs after administration, which is a good predictor of carrier’s in vivo toxicity^65^. These results indicated that the GANPs can be used as a biocompatible and non-toxic agent and an efficient antibacterial agent with the potential to ameliorate the problems associated with development of the multi-drug-resistant bacteria. These findings agreed with the reports from Nimi et al. who showed that there is no aggregation of blood cells (WBCs, RBCs, and Platelets) after incubation with AuNPs at the given concentration of 1 mg, were in the hemolysis induced by the AuNPs at 0.1%^66^.

**Table 2:** Display of the human blood components treated with 1 mg/ml of GANPs.

Blood components	Control	With GANP	Unit
White blood cells	5.29	5.26	× 10^3^/µL
Lymphocyte	31.07	30.6	%
Monocyte	8.91	9.19	%
Neutrophil	58.72	58.72	%
Eosinophil	0.97	1.1	%
Basophil	0.33	0.39	%
Red blood cells	5.51	5.44	× 10^6^/µL
Hemoglobin	15.01	15.03	g/dL
Hematocrit	46.8	46.4	%
Platelets	171.7	158.8	× 10^3^/µL

## Conclusions

Acne vulgaris is considered one of the most chronic inflammatory skin diseases, which can lead to profound long-term alterations of skin, especially of the face, even after treatment and cure. It affects not only the physical health but also the psychological, and overall quality of life of the patient. Different types of microorganisms cause acne, especially gram-positive bacteria, which have shown several virulence factors, and antibiotic resistance. *C. innocuum,* which is the most commonly found and isolated anaerobic bacteria from acne samples, followed by other bacteria, e.g., *L. plantarum*, *A. prevotii, and P. asaccharolyticus* cause the condition. A majority of anaerobic bacteria are difficult to treat because of their ability to produce biofilm, toxins, spores, and multi-drug resistance. The findings of the present study revealed that the chemical reduction method of HAuCl_4_ by sodium alginate to produce the gold-nanoparticles cored nanopreparation (GANPs) which were characterized by their physicochemical properties using UV–Vis, SEM, FT-IR, XRD, and EDX were also biocompatible. The GANPs demonstrated excellent inhibition of bacterial growth, low dose MIC, anti-adhesion, and anti-biofilm activities which confirmed their significant antibacterial activity at low concentrations against these gram-positive acne-based anaerobic bacterial strains. The utilization of non-antibiotic, alternative acne treatments, such as the GANPs may diminish the resistance rates of the bacterial strain isolates found in this study. Furthermore, the results also confirmed the GANP’s hemocompatibility in white and red blood cells, as well as platelets. The remarkable biological activity of the antimicrobial nanopreparation presented significant potential for effective treatment of acne in clinical settings, as part of the newer therapy.

## Data Availability

All data are included in the article.
